# A Comprehensive Review of the Role of Magnesium in Critical Care Pediatrics: Mechanisms, Clinical Impact, and Therapeutic Strategies

**DOI:** 10.7759/cureus.66643

**Published:** 2024-08-11

**Authors:** Accha Nandini Sagar, Vishal Kalburgi, Jayant D Vagha, Amar Taksande, Revat J Meshram, Sham Lohiya

**Affiliations:** 1 Department of Pediatrics, Jawaharlal Nehru Medical College, Datta Meghe Institute of Higher Education and Research, Wardha, IND

**Keywords:** clinical impact, therapeutic strategies, magnesium deficiency, pediatrics, critical care, magnesium

## Abstract

Magnesium is an essential mineral with pivotal roles in various physiological processes, including enzyme function, neuromuscular regulation, and cardiovascular health. Magnesium's importance in critically ill pediatric patients is magnified due to its involvement in maintaining cellular homeostasis and potential therapeutic benefits. This review comprehensively analyzes magnesium's role in critical care pediatrics, focusing on its physiological mechanisms, clinical impact, and therapeutic strategies. Magnesium’s functions in energy production, protein synthesis, and electrolyte balance underscore its significance in critical illness, where imbalances can lead to severe complications such as arrhythmias, neuromuscular disturbances, and respiratory issues. The review examines the clinical consequences of magnesium deficiency, including its impact on various body systems and the potential exacerbation of critical conditions. It also explores therapeutic strategies to optimize patient care, including supplementation practices, dosing considerations, and monitoring protocols. By summarizing recent research and clinical guidelines, this review aims to enhance understanding of magnesium's role in critical care and provide evidence-based recommendations for its management. The insights provided are intended to guide clinicians in integrating magnesium therapy into critical care practices, ultimately improving patient outcomes and advancing the management of critically ill pediatric patients.

## Introduction and background

Magnesium is a vital mineral that underpins many essential physiological processes in the human body. It is a cofactor for more than 300 enzymatic reactions and is crucial for energy production, protein synthesis, and cellular metabolism [1]. Magnesium plays a central role in regulating neuromuscular function, contributing to the proper functioning of muscles and nerves. It is integral to maintaining cellular homeostasis by stabilizing cell membranes and facilitating the synthesis of nucleic acids [2]. Additionally, magnesium is crucial for cardiovascular health, helping to regulate heart rhythm and support overall cardiac function. Its role extends to maintaining bone health, modulating immune responses, and ensuring the balance of other electrolytes, such as calcium and potassium, which is essential for systemic equilibrium [3].

In the context of critical care pediatrics, the importance of magnesium becomes even more pronounced. Critically ill children often experience disrupted magnesium homeostasis due to a combination of factors, including increased renal or gastrointestinal losses, altered nutritional intake, and the heightened metabolic demands of their illness [4]. This disruption can lead to significant complications, such as arrhythmias, neuromuscular disturbances, and impaired respiratory function. Magnesium deficiency or imbalance may exacerbate the severity of these conditions, highlighting the need for careful monitoring and management [5]. On the other hand, magnesium supplementation has emerged as a potentially beneficial therapeutic strategy in various critical conditions, such as sepsis, respiratory distress, and seizures. Given the complex nature of critical illness, understanding the nuanced role of magnesium is crucial for optimizing patient care and improving outcomes [5].

This review aims to provide an in-depth analysis of magnesium's role in critical care pediatrics by examining its physiological mechanisms, clinical implications, and therapeutic applications. The objectives are threefold: first, to elucidate the physiological functions of magnesium and its critical roles in maintaining health; second, to evaluate the clinical impact of magnesium deficiency in critically ill pediatric patients, including its effects on various body systems; and third, to review current therapeutic strategies involving magnesium, focusing on supplementation practices, dosage considerations, and potential side effects. Additionally, the review will summarize recent research findings and clinical guidelines to offer evidence-based recommendations for integrating magnesium management into critical care practices. Through this comprehensive exploration, the review aims to enhance clinical understanding and guide effective magnesium use in improving patient outcomes in critical care settings.

## Review

Magnesium physiology and metabolism

Magnesium is an essential mineral critical in numerous cellular functions within the body. It is a vital cofactor for over 300 enzymatic reactions, fundamental to energy production, protein synthesis, and nucleic acid synthesis. Magnesium influences enzyme activity by binding to ligands such as adenosine triphosphate, facilitating enzyme conformational changes, and promoting multienzyme complexes aggregation [6]. Additionally, magnesium competes with calcium for binding sites on cell membranes, which is crucial for maintaining the electrical properties of these membranes. This competition helps regulate intracellular calcium concentrations, affecting various cellular functions, including muscle contraction, neurotransmitter release, and cellular signaling pathways [7]. The regulation of magnesium homeostasis is a complex process involving intestinal absorption, exchange with bone, and renal excretion. Approximately 20% of filtered magnesium is reabsorbed in the proximal tubule of the kidneys, while about 60% is reclaimed in the cortical thick ascending limb (TAL). The remaining 5%-10% is reabsorbed in the distal convoluted tubule (DCT) [8]. TAL's absorption is primarily passive and paracellular, potentially influenced by mutations in proteins like claudin-16, associated with familial hypomagnesemia with hypercalciuria and nephrocalcinosis. In contrast, active transcellular transport in the DCT is regulated by transient receptor potential melastatin 6 channels, significantly controlling urinary magnesium excretion [9]. In terms of distribution, magnesium is predominantly stored in the body's bones, comprising approximately 60% of the total body magnesium. About 31% is located within cells, while only 2% is in serum. Intracellular magnesium is maintained within narrow concentration limits, which is crucial for cellular function, especially during conditions of stress such as hypoxia or prolonged depletion [10]. Serum magnesium levels are tightly regulated, with the kidneys being the primary organ responsible for maintaining this balance. This intricate system ensures that magnesium levels remain stable, supporting its essential roles in cellular physiology and overall health [11].

Mechanisms of magnesium in critical illness

Magnesium is essential for proper neuromuscular excitability and the transmission of neural impulses. It is a critical cofactor for various enzymatic reactions and is key in regulating neurotransmitter release at the neuromuscular junction. Low magnesium levels, as seen in hypomagnesemia, can lead to significant neuromuscular complications, including respiratory muscle weakness and bronchospasm [12]. These conditions may increase the need for mechanical ventilation in critically ill patients. Conversely, hypermagnesemia can result in flaccid muscle paralysis and hyporeflexia, further complicating the clinical situation and potentially leading to respiratory depression [13]. Magnesium also plays a vital role in maintaining cardiac function and stability. As a natural calcium antagonist, it helps regulate vascular tone and myocardial contractility without causing myocardial depression. Magnesium administration can enhance cardiac output, compensating for its vasodilatory effects and helping prevent hypotension. Additionally, magnesium is effective in treating specific arrhythmias, such as torsade de pointes and those induced by digitalis toxicity. By stabilizing cardiac electrical activity, magnesium can significantly reduce the risk of life-threatening arrhythmias in critically ill patients [14]. Magnesium's role in respiratory function is particularly noteworthy in critical illness. Magnesium deficiency is linked to respiratory muscle weakness and increased bronchospasm, elevating the risk of acute respiratory failure and the need for mechanical ventilation. While the exact impact of correcting magnesium levels on the development of respiratory failure remains an area of ongoing research, it is evident that maintaining adequate magnesium levels is crucial for optimal respiratory function in critically ill patients [15]. Magnesium does not act in isolation; it closely interacts with other electrolytes, particularly calcium and potassium. In critically ill patients, hypomagnesemia often coexists with hypocalcemia and hypokalemia, creating a complex interplay that can exacerbate clinical conditions. Studies have shown that a significant percentage of patients with low magnesium levels also present with normal calcium and potassium levels, indicating that magnesium status should be carefully monitored alongside other electrolytes to ensure comprehensive management [11]. Moreover, magnesium has significant immunomodulating capabilities, crucial to the body's inflammatory and immune responses. Hypomagnesemia has been associated with an increased risk of infections, exacerbation of sepsis progression, and decreased survival rates in critically ill patients. Supplementation with magnesium has demonstrated benefits in modulating the inflammatory response, potentially improving outcomes in this population. By supporting immune function and reducing inflammation, magnesium can be a valuable component of therapeutic strategies in critical care settings [4].

Clinical impact of magnesium deficiency in critical care pediatrics

**Figure 1:**
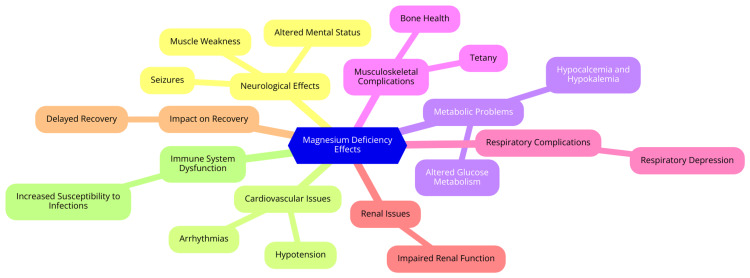
The clinical impacts of magnesium deficiency in critical care pediatrics Image credit: Accha Nandini Sagar

Magnesium deficiency is a significant concern in critical care pediatrics, impacting various physiological systems and clinical outcomes. Understanding magnesium deficiency's common causes, clinical manifestations, diagnostic approaches, and systemic impacts is essential for effectively managing critically ill children [5]. Magnesium deficiency can arise in critically ill children due to several factors. One of the primary causes is increased magnesium losses due to renal dysfunction, which is prevalent in many critically ill patients. Additionally, gastrointestinal losses, such as diarrhea, can exacerbate magnesium depletion [16]. Diuretics, commonly administered in critical care settings, can also increase urinary magnesium excretion. Inadequate dietary intake is another contributing factor, especially in patients who cannot eat or receive parenteral nutrition without sufficient magnesium supplementation. Furthermore, certain conditions, such as sepsis, trauma, and burns, can elevate the metabolic demand for magnesium or disrupt its homeostasis, leading to deficiency rates as high as 65% in critically ill pediatric patients [17]. The clinical manifestations of magnesium deficiency in critically ill children can be diverse and severe. Neurologically, patients may present with symptoms such as muscle weakness, seizures, and increased neuromuscular excitability, leading to tetany and tremors [18]. Cardiovascularly, magnesium deficiency is associated with dysrhythmias, increased heart rate, and hypertension, highlighting its crucial role in cardiac function. Musculoskeletal symptoms can include muscle cramps and spasms due to impaired neuromuscular transmission. General symptoms such as fatigue, irritability, and altered mental status may also indicate magnesium deficiency, complicating the overall clinical picture [19]. To diagnose magnesium deficiency, serum magnesium levels are typically measured, with levels below 1.5 mEq/L indicating hypomagnesemia. However, serum levels may not accurately reflect total body magnesium stores, necessitating a comprehensive clinical assessment. A thorough evaluation, including patient history and physical examination, is essential to identify risk factors and symptoms associated with deficiency. It is also important to assess levels of other electrolytes, as hypomagnesemia often coexists with hypocalcemia and hypokalemia, further complicating the clinical management of critically ill patients [20]. The impact of magnesium deficiency extends across multiple physiological systems. In the cardiovascular system, magnesium is vital for maintaining normal heart rhythm, and its deficiency can increase the risk of arrhythmias and elevated blood pressure, complicating patient management. Neurologically, magnesium deficiency can result in increased neuromuscular excitability, manifesting as seizures, muscle spasms, and altered mental status, which can significantly affect patient care and recovery. Magnesium is critical for muscle function; deficiency can lead to muscle weakness and cramps, impacting mobility and overall rehabilitation, particularly in patients requiring mechanical ventilation or neuromuscular disorders [19]. The clinical impacts of magnesium deficiency in critical care pediatrics are illustrated in Figure 1.

Therapeutic strategies

Magnesium supplementation in critical care pediatrics is essential for managing various clinical conditions. It plays a vital role in addressing hypomagnesemia, a common issue among critically ill patients that can lead to severe complications, including cardiac arrhythmias and increased mortality rates [21]. Additionally, magnesium is indicated for managing severe asthma exacerbations, where it can help improve lung function and reduce hospital admissions. It also effectively treats cardiac arrhythmias, particularly conditions like torsades de pointes and rapid atrial fibrillation. Furthermore, magnesium may benefit patients with neuromuscular disorders and those experiencing sepsis or inflammatory states, given its immunomodulatory effects [21]. The administration of magnesium can be tailored to the clinical scenario and urgency of treatment. Intravenous (IV) administration is commonly used in acute settings for rapid correction of hypomagnesemia or during severe asthma attacks. Typical dosages often start at 1-2 g administered intravenously for 15-30 minutes, allowing for a quick response in critical situations [22]. Oral supplementation is another option, particularly for maintenance therapy or less acute cases, with dosages typically ranging from 200 to 400 mg daily, depending on the patient's needs and tolerance. In some instances, a continuous IV infusion may be necessary to maintain adequate magnesium levels, especially in patients with ongoing losses or high requirements [23]. Monitoring is crucial to ensure the safety and efficacy of magnesium therapy. Regular monitoring of serum magnesium levels is essential, particularly in patients with renal impairment or those receiving high doses of magnesium. Clinicians should also observe for clinical signs of magnesium deficiency, such as muscle cramps and arrhythmias, as well as signs of toxicity, including respiratory depression and hypotension. Renal function must be considered, as patients with renal dysfunction may require adjustments in magnesium dosages based on their serum levels and overall renal function [6]. While magnesium therapy is generally safe, it is not without potential complications. Hypermagnesemia can occur, particularly in patients with renal failure, leading to symptoms such as hypotension, respiratory depression, and even cardiac arrest. Neuromuscular effects may also arise from elevated magnesium levels, including muscle weakness and reduced reflexes. Furthermore, magnesium can interact with certain medications, such as diuretics and antibiotics, potentially affecting their absorption and efficacy. Gastrointestinal disturbances, such as diarrhea or discomfort, may occur with oral magnesium supplementation in some patients [24].

Evidence-based clinical applications

Magnesium's role in managing critical conditions in pediatric patients has gained increasing recognition recently. Its applications are particularly significant in conditions such as sepsis, acute respiratory distress syndrome (ARDS), neurological disorders (including seizures), and cardiac arrhythmias. Understanding the evidence-based clinical applications of magnesium in these contexts is essential for optimizing patient care in critical settings [25]. In the context of sepsis, magnesium deficiency has been linked to an increased susceptibility to infections and a higher risk of developing septic shock. Retrospective studies have shown that hypomagnesemia is associated with an elevated risk of sepsis, with odds ratios indicating a strong correlation. Furthermore, recent cohort studies have demonstrated that administering magnesium sulfate can improve lactate clearance in septic patients, suggesting its potential therapeutic role. Notably, magnesium sulfate use has been associated with lower 28-day all-cause mortality in critically ill patients with sepsis, highlighting its protective effects [26]. Magnesium is also crucial in managing ARDS. Hypomagnesemia can lead to respiratory muscle weakness, exacerbating conditions like ARDS. While specific studies on magnesium's direct effects on ARDS in pediatric patients are limited, its role in modulating inflammatory responses suggests potential benefits in managing this condition. Maintaining adequate magnesium levels may support respiratory function and reduce the incidence of respiratory failure requiring mechanical ventilation [27]. Magnesium's neuroprotective properties in neurological disorders are particularly important, especially in managing seizures. Magnesium sulfate is commonly used in pediatric emergency settings for treating seizures, including those associated with conditions like eclampsia and torsades de pointes. Research supports its efficacy in reducing seizure frequency and improving outcomes in critically ill children. Additionally, magnesium's role in protecting neuronal integrity during hypoxic events may contribute to better outcomes for pediatric patients with neurological disorders [28]. Magnesium is essential for maintaining normal cardiac function, particularly in managing arrhythmias. In cases of torsades de pointes, magnesium sulfate is considered a first-line treatment, as its administration can stabilize cardiac rhythms and prevent life-threatening arrhythmias in critically ill children. Clinical evidence indicates that magnesium supplementation can reduce the incidence of arrhythmias in critically ill patients, underscoring its importance in cardiac care [29]. Recent literature consistently emphasizes the significance of magnesium in critical care settings. Studies have shown that magnesium levels correlate with sepsis outcomes, with hypomagnesemia linked to higher mortality rates. Randomized controlled trials have demonstrated that magnesium supplementation can improve lactate clearance and reduce ICU length of stay in septic patients. However, there is a pressing need for more prospective studies to elucidate further magnesium's role in critical care, particularly regarding optimal dosing and timing of supplementation [30].

Guidelines and recommendations

Clinical guidelines and recommendations regarding magnesium use in critical care pediatrics are essential for optimizing patient outcomes. Several organizations have established guidelines that underscore the importance of magnesium in managing critically ill pediatric patients. For instance, the American Heart Association recommends administering magnesium sulfate at a dose of 50 mg/kg (maximum 2 g) for conditions such as torsades de pointes and severe asthma exacerbations, to be infused over 10 minutes. This guideline highlights the critical role of timely magnesium treatment in life-threatening situations. The National Asthma Education and Prevention Program also includes magnesium sulfate as an adjunct therapy for severe asthma exacerbations, particularly in emergency settings, supporting its efficacy in improving respiratory function [31]. Given the high prevalence of magnesium imbalances in critically ill pediatric patients, regularly monitoring serum magnesium levels is advised. Clinicians should assess magnesium levels upon admission to the pediatric intensive care unit and continue monitoring throughout the patient's stay [32]. Hypomagnesemia has been associated with increased mortality rates and prolonged ICU stays, making vigilant monitoring imperative. Furthermore, IV magnesium sulfate should be administered to patients identified with hypomagnesemia according to established dosing guidelines. Treatment should be tailored based on the clinical scenario, considering factors such as the patient's overall condition and any concurrent electrolyte abnormalities [32]. Integrating magnesium therapy into the overall care plan for critically ill children is crucial, especially for those with conditions like asthma or cardiac arrhythmias. Clinicians must be aware of the potential adverse effects of both hypomagnesemia and hypermagnesemia, ensuring that magnesium administration is effective and safe. Additionally, ongoing education for healthcare providers about the significance of magnesium in critical care settings is vital. This education should encompass understanding the physiological roles of magnesium, recognizing signs of deficiency, and knowing the appropriate therapeutic interventions [4].

Future directions

Future research on magnesium in critical care pediatrics should focus on several key areas, potential advancements in therapy, and emerging technologies for supplementation. A deeper mechanistic understanding is crucial, as more studies are needed to elucidate the precise mechanisms by which magnesium influences critical physiological processes, particularly in pediatric populations. This includes investigating how magnesium affects intracellular signaling pathways and its role in modulating immune responses [33]. Clinical outcomes are another important focus. Research should explore the relationship between magnesium levels and clinical outcomes in critically ill children, including the impact of magnesium supplementation on mortality rates, length of ICU stays, and recovery times from critical illnesses. Determining the optimal dosage and administration is also a key consideration. There is a need for randomized controlled trials to establish the best dosage, timing, and route of magnesium administration (oral vs. IV) for different clinical scenarios, such as arrhythmias and electrolyte imbalances. Longitudinal studies are essential to assess the long-term effects of magnesium supplementation and its potential role in preventing chronic conditions associated with critical illness, such as cardiovascular diseases and osteoporosis [34]. Potential advancements in magnesium therapy and monitoring could include the development of standardized protocols for magnesium monitoring and supplementation in pediatric ICUs, enhancing consistency in treatment and improving patient outcomes. These protocols should be informed by evidence from ongoing research. Advances in genetic and metabolic profiling may allow for personalized magnesium therapy, tailoring supplementation based on individual patient needs and responses. Exploring the synergistic effects of magnesium with other treatments, such as vitamin D and calcium supplementation, could enhance therapeutic efficacy in managing conditions like osteoporosis and cardiovascular health in critically ill children [35]. Emerging technologies and innovations in magnesium supplementation hold great promise. Innovations in nanotechnology could lead to the development of more effective magnesium delivery systems, enhancing absorption and bioavailability in pediatric patients. This could include nanoencapsulated magnesium supplements that improve stability and reduce gastrointestinal side effects [36]. The integration of wearable technology and smart monitoring devices could facilitate real-time tracking of magnesium levels in patients, allowing for timely interventions and adjustments in therapy. Additionally, research into biodegradable magnesium-based implants for orthopedic applications may provide new treatment avenues for pediatric patients, particularly in managing fractures and bone health. Promoting dietary sources of magnesium through innovative food products or supplements could improve overall magnesium intake in children, especially in populations at risk of deficiency [36].

## Conclusions

In conclusion, magnesium is a vital mineral for maintaining health, particularly in critical care pediatrics, due to its significant roles in neuromuscular function, cardiovascular health, and cellular metabolism. Imbalances in magnesium can lead to severe complications such as arrhythmias, neuromuscular disturbances, and respiratory issues in critically ill children. Effective management through monitoring and supplementation can improve clinical outcomes and support recovery. Ongoing research and integration of emerging findings into clinical practice are essential to optimize magnesium therapy and enhance patient care in critical care settings.
